# Effects of intravitreal insulin and insulin signaling cascade inhibitors on emmetropization in the chick

**Published:** 2012-10-20

**Authors:** Alexandra Marcha Penha, Eva Burkhardt, Frank Schaeffel, Marita P. Feldkaemper

**Affiliations:** University Eye Hospital, Institute for Ophthalmic Research, Section of Neurobiology of the Eye, Tuebingen, Germany

## Abstract

**Purpose:**

Intravitreal insulin has been shown to be a powerful stimulator of myopia in chickens, in particular if the retinal image is degraded or defocused. In most tissues, the insulin receptor activates two main signaling pathways: a) the mitogen-activated protein kinase (MAPK) cascade (e.g., mitogen-activated protein kinasem kinase [MEK] and extracellular regulated kinase [ERK]) and b) the phosphatidylinositol 3-kinase (PI3K)/protein kinase B (Akt) pathway. In the current study, insulin was injected, and these pathways were separately inhibited to determine which is activated when the retinal image is defocused by spectacle lenses.

**Methods:**

Chicks were treated with either +7 D, −7 D, or no lenses. They were intravitreally injected with insulin, the MEK inhibitor U0126, the PI3K inhibitor Ly294002, or a combination of insulin and one of the inhibitors. Refractions and ocular dimension were measured at the beginning and after four days of treatment. The retinal proteins of the chicks were measured with western blots after 2 h and four days of treatment. Incubation occurred with anti-Akt1, anti-Erk1/2, anti-phospho-Akt^Thr308^, and anti-phospho-Erk1/2^(Thr202/Tyr204)^ antibodies, and the ratio between the relative intensity of the phospho-form and the total-form was calculated.

**Results:**

Chicks wearing positive lenses and injected with saline and with PI3K inhibitor compensated for the imposed defocus and became hyperopic. Insulin injections and insulin plus PI3K inhibitor injections prevented lens-induced hyperopia, whereas the MEK inhibitor alone and insulin plus MEK inhibitor had no effect. Obviously, the MEK inhibitor suppressed the effect of insulin on eye growth in the plus lens–treated animals. Chicks treated with negative lenses and injected with insulin, or with insulin plus MEK inhibitor, overcompensated for the imposed defocus. This effect of insulin was not detected in eyes injected with PI3K inhibitor plus insulin, suggesting that the PI3K inhibitor suppressed the effects of insulin in minus lens–treated animals. Insulin increased the ratio of phospho-Akt/total-Akt in animals with normal visual exposure but even more so in chicks wearing plus or minus lenses. The increase was blocked by simultaneous PI3K inhibitor injections in control eyes but not in lens-treated eyes. Insulin also increased the ratio of phospho-ERK/total-ERK in animals with normal visual exposure and in animals wearing positive lenses, compared to U0126- and Ly294002-injected eyes. In contrast, no significant activation of the MEK/ERK pathway was observed in the negative lens–treated animals.

**Conclusions:**

Intravitreal insulin promoted axial eye growth and stimulated both signaling pathways. The PI3K/Akt pathway was activated in control and plus and minus lens–treated eyes, but the MEK/ERK pathway was activated only with positive lenses or no lenses. With negative lenses, insulin did not stimulate the MEK/ERK signaling cascade. Independent of the pathway stimulated after insulin binding, the effect on insulin was always the same: an increase in eye growth.

## Introduction

According to the World Health Organization (WHO), the most common causes of visual impairments are uncorrected refractive errors, such as myopia, hyperopia, or astigmatism, followed by cataract and glaucoma [1]. Animal models of myopia have been developed and have shown that emmetropization in the vertebrate eye is guided by an active, visually guided feedback loop [2]. Animals compensate for imposed defocus by adjusting the axial eye growth rate such that the focal plane and the photoreceptor plane achieve a close match. Regulation of eye growth was demonstrated to be largely independent of processing in the brain, as demonstrated in optic nerve lesion studies [3-7].

Several retinal substances were shown to be implicated in visually guided eye growth regulation, such as vasoactive intestinal peptide (VIP) [8,9], dopamine [10-12], retinoic acid [13-15], glucagon [16-18], insulin [19,20], γ-aminobutyric acid (GABA) [21], and growth factors such as transforming growth factor (TGF) [22,23], basic fibroblast growth factor (bFGF) [22], and insulin-like growth factor-1 (IGF-1) [20]. Moreover, experiments in chickens and mice have implicated the early growth response gene-1 (Egr-1, also called ZENK in chickens) [24-26] in the feedback mechanisms for visual control of axial eye growth and myopia development. However, the mechanism and the signaling pathways are not yet known. Because some of these modulators were found to be upregulated under conditions that inhibit eye growth, they were considered stop signals, like glucagon and ZENK, in the chicken model [16,27]. Glucagon and insulin have opposite effects on metabolic functions in the body, on cell proliferation in progenitor cells at the periphery of the retina [28], and on axial eye growth [19,20]. While intravitreal injections of glucagon or a glucagon agonist can prevent negative lens–induced myopia in chicks, by slowing axial eye growth and increasing choroidal thickness [16,20,29], insulin not only blocks the development of hyperopia, which is normally induced by positive lenses, but also induces axial myopia. In addition, insulin enhances myopia induced by negative lens treatment [19]. Insulin and IGF-1 induce axial myopia even in eyes not wearing any lenses [20].

Insulin binds and acts mainly through the insulin receptor but can also act via the IGF-1 receptor. In addition, IGF-1 can bind to the insulin receptor. In recent years, several studies investigated IGF-1 signal transduction cascades involved in neuroprotection. However, whether the same pathways are activated upon insulin or IGF-1 receptor stimulation during eye growth is currently unknown. Insulin and IGF-1 can mediate their cellular effects by signaling downstream through insulin receptor substrate (IRS) molecules. Ligand binding to the corresponding receptors leads to conformational changes and induces a cascade of phosphorylation of several cytosolic molecules [30]. Depending on the phosphorylated downstream molecules, different pathways are activated. The MEK/ERK pathway involves binding of the tyrosine phosphorylated (TP)-IRS to the growth factor receptor-bound protein 2 (Grb2), which results in sequential activation of p21^Ras^, mitogen-activated protein kinase (MEK), and mitogen-activated protein kinase (MAPK originally called ERK) [31]. ERK activation directly contributes to insulin- and IGF-1-stimulated mitogenesis, neuritic development, and gene expression [32-34], control of the cell cycle and cell migration, as well as proliferation and differentiation. In addition to this pathway, various cellular stimuli, including activation of the insulin receptor and the IGF-1 receptor, can activate the PI3K pathway. The serine/threonine kinase Akt is a crucial kinase in this pathway. PI3K phosphorylates PI(4,5)P2, generating PI(3,4,5)P3, a potent second messenger required for survival signaling and insulin action [35]. The PI3K pathway controls glucose metabolism [36,37]. This includes glucose uptake and glycogenesis. Furthermore, fundamental cellular functions such as transcription, translation, proliferation, growth, and survival are regulated [38,39]. Generally, the mitogenic signal of the IGF-1 receptor/protein interaction is promoted through the PI3K/Akt pathway, but sometimes is transmitted via either the MEK/ERK pathway or mitochondrial translocation of Raf-1. IGF-1 has been shown to activate the MEK/ERK and PI3K/Akt pathways to induce cellular transformation [40]. The PI3K/Akt pathway often transduces signals that are similar in nature to that of the MEK/ERK pathway, and several studies reported a cross talk between those two pathways on multiple levels [40]. Contrary to mammals, which have two isoforms of ERK, called p44 ERK1 and p42 ERK2, the chick has only one isoform, the p44 ERK2 [41]. It is not known whether the lack of one isoform determines any differences at the cell signaling level [42].

In the nervous system, most in vitro studies showed that members of the insulin family, especially IGF-1, act as a neuroprotective factor [43-45], and require an initial activation of PI3K as a common step in their intracellular signaling pathway for survival effects. Proinsulin and insulin, before IGF-1, act as survival factors in the chick and mouse retina during development [46-49]. Furthermore, it was shown that proinsulin can act independently of Akt activation [49]. In the embryonic stages, at E5, the insulin survival effects are mediated through MEK/ERK activation, whereas the same effect is mediated through the PI3K/Akt activation at embryonic stage E9 [50].

The role of insulin receptor signaling in neuronal tissues has not been characterized in detail. A recent study [51] suggested that different ocular layers express different insulin receptor transcripts, suggesting that the same receptor probably can activate different signaling cascades, which then depend not only on the stimuli but also on the tissue. The present study analyzed the effect of insulin intravitreal injections on the activation of the PI3K/Akt or MEK/ERK signaling cascades as a possible mechanism responsible for eye growth in the chicken model of myopia.

## Methods

### Treatment of animals

Eight-day-old male White Leghorn chickens (n=108) were raised under a 12 h:12 h light-dark cycle. To attach the lenses, Velcro rings were glued onto the feathers around the eyes a few hours before the lens treatment was started. Animals were anesthetized before intravitral injections by exposure to diethyl ether by inhalation. Because insulin binding to the insulin receptor was shown to activate the MEK/ERK pathway and the PI3K/Akt- pathway, selective inhibitors were used to separate the inputs. U0126 was used to block the activation of ERK 1/2 that occurs by inhibiting the activity of MEK. This effect is mediated by antagonizing the AP-1 transcriptional function via noncompetitive inhibition of the dual specificity kinase MEK with a half maximal inhibitory concentration (IC_50_) of 0.07 μM for MEK1 and 0.06 μM for MEK2 [52]. Ly294002 was used to inhibit PI3-kinases. Ly294002 is a potent and specific cell-permeable inhibitor of PI3K with IC_50_ values in the range of 1–50 μM, and competitively inhibits adenosine 5′-triphosphate binding to the catalytic subunit of PI3-kinases [53]. The inhibition of PI3K in turn leads to a reduction in the phosphorylation of Akt, as shown in previous studies [50]. The experimental treatment was in accordance with the ARVO Statement for Care and Use of Animals in Ophthalmic and Vision Research and was approved by the University Commission for Animal Welfare. The details of all the treatment groups are summarized in Table 1.

**Table 1:** Summary of experimental plan.

Group number	Binocular lens treatment	Ipsilateral eye injection (12.5 µl)	Contralateral eye injection (12.5 µl)	Concentration [µM] ips. eye/contra. eye	Time of treatment
**Effects of lens wear and intravitreal insulin, MEK- (U0126) and PI3K- (Ly294002)-inhibitor injections on refractive development and axial length**					
Group 1	Without lenses	Saline	Insulin	0/0.3	4 days
Group 2	Without lenses	U0126	U0126+insulin	50/50+0.3	4 days
Group 3	Without lenses	Ly294002	Ly294002+insulin	50/50+0.3	4 days
Group 4	Positive lenses (+7D)	Saline	Insulin	0/0.3	4 days
Group 5	Positive lenses (+7D)	U0126	U0126+insulin	50/50 + 0.3	4 days
Group 6	Positive lenses (+7D)	Ly294002	Ly294002+insulin	50/50 + 0.3	4 days
Group 7	Negative lenses (−7D)	Saline	Insulin	0/0.3	4 days
Group 8	Negative lenses (−7D)	U0126	U0126+insulin	50/50+0.3	4 days
Group 9	Negative lenses (−7D)	Ly294002	Ly294002+insulin	50/50+0.3	4 days
**Effects of intravitreal insulin, MEK- (U0126) and PI3K- (Ly294002) inhibitor injections on phosphorylation levels**					
Group 10	Without lenses	Saline	Insulin	0/0.3	2 h
Group 11	Without lenses	U0126	Ly294002	100/100	2 h
Group 12	Without lenses	U0126	U0126+insulin	50/50+0.3	2 h
Group 13	Without lenses	Ly294002	Ly294002+insulin	50/50+0.3	2 h
Group 14	Positive lenses (+7D)	Saline	Insulin	0/0.3	2 h
Group 15	Positive lenses (+7D)	U0126	Ly294002	100/100	2 h
Group 16	Positive lenses (+7D)	U0126	U0126+insulin	50/50+0.3	2 h
Group 17	Positive lenses (+7D)	Ly294002	Ly294002+insulin	50/50+0.3	2 h
Group 18	Negative lenses (−7D)	Saline	Insulin	0/0.3	2 h
Group 19	Negative lenses (−7D)	U0126	Ly294002	100/100	2 h
Group 20	Negative lenses (−7D)	U0126	U0126+insulin	50/50+0.3	2 h
Group 21	Negative lenses (−7D)	Ly294002	Ly294002+insulin	50/50+0.3	2 h

### Effects of intravitreal insulin and U0126 and Ly294002 injections on refractive development and axial length

At the age of 8 days post-hatching, chicks were intravitreally injected (12.5 µl) in both eyes (Table 1).

Injections were either saline in one eye and insulin (0.3 µM Insuman Rapid, Aventis, Frankfurt, Germany, groups 1, 4, and 7) in the contralateral eye, or U0126 (50 µM, Calbiochem) in one eye and a combination of insulin (0.3 µM) and the inhibitor U0126 (50 µM; groups 2, 5, and 8) in the contralateral eye, or Ly294002 (50 µM, Calbiochem) in one eye and a combination of insulin (0.3 µM) and the inhibitor Ly294002 (50 µM; groups 3, 6, and 9) in the contralateral eye. All types of injections were repeated two days later. In addition, from day 8 until the end of the treatment (day 12), chicks were binocularly treated with either positive lenses (groups 4 to 6) or negative lenses (groups 7 to 9), or the chicks did not wear any lenses (groups 1 to 3). Before the injections and the beginning of the lens treatment and four days later, the refractive state and axial length were measured. Six animals were treated per group.

### Short- and long-term effects of lens wear and intravitreal injections of insulin, U0126, and Ly294002 on extracellular regulated kinase 1/2 and protein kinase B phosphorylation levels

Signaling cascades triggering myopia or hyperopia onset may differ from those maintaining their progression. Therefore, two different time points were investigated. For the analyses of long-term effect (four days) of lens wear and intravitreal injections of insulin, MEK inhibitor, and PI3K inhibitor, the retinas from groups 1 to 9 (Table 1) were dissected, and the extracted proteins were subjected to western blot analysis. To investigate the short-term effects (2 h) of lens wear and intravitreal injections on ERK1/2 and Akt phosphorylation levels, animals (six per group) were treated for 2 h with no lens (groups 10 to 13), positive lenses (groups 14 to 17), or negative lenses (groups 18 to 21) in combination with the following injections (12.5 µl): saline in one eye and insulin (0.3 µM) in the contralateral eye (groups 10, 14, and 18), U0126 (100 µM) in one eye and Ly294002 (100 µM) in the contralateral eye (group 11, 15, 19), U0126 in one eye and insulin plus U0126 in the contralateral eye (group 12, 16, and 20), and Ly294002 in one eye and a combination of insulin (0.3 µM) and the inhibitor Ly294002 (50 µM) in the other eye (groups 13, 17, and 21; Table 1).

### Measurements of refractive state and ocular dimensions

Refractive states were measured with automated infrared photoretinoscopy [54]. Axial length, defined as the distance from the surface of the cornea to the vitreoretinal interface, was measured with A-scan ultrasonography [55]. This technique also provided data on anterior chamber depth, lens thickness, and vitreous chamber depth.

### Tissue preparation

Chicks were euthanized with an overdose of diethylether by inhalation. Eyes were enucleated and vertically cut with a razor blade, discarding the anterior part containing the lens. The vitreous body was removed and the pecten cut out. From the posterior pole of the eye, a biopsy punch of 8 mm was excised and placed in a Petri dish filled with ice-chilled saline. The retinas from all experimental groups were separated from the rest of the fundal layers under visual control under a dissecting microscope, and immediately frozen in liquid nitrogen and stored at −80 °C until protein extraction.

### Total protein extraction

Retinal total protein extraction was performed using T-PER Tissue Protein Extraction Reagent (Thermo Scientific, Pierce Biotechnology, Rockford, IL), according to the manufacturer’s instructions. To prevent phosphatase action and proteolytic degradation 1 × PhosSTOP Phosphatase Inhibitor Cocktail (Roche, Mannheim, Germany) and 1 × of complete Protease Inhibitor Cocktail (Roche) were added to the extraction buffer. The total protein amount was measured using the Pierce BCA Protein Assay kit (Thermo Scientific, Pierce Technology).

### Western blot analysis

Ten micrograms of protein were mixed with Laemmli buffer, heated at 95 °C for 4 min, fractionated by 8% sodium dodecyl sulfate–PAGE and transferred to Trans-Blot Medium Pure Nitrocellulose membranes (Bio-Rad Laboratories, Hercules, CA). The membranes were blocked overnight at 4 °C with 3% BSA in Tris-buffered saline with 1% Tween-20 (TBST; Roth, Karlsruhe, Germany). The next day, membranes were incubated for 1 h at room temperature with the following primary antibodies: goat anti-Akt1 (1:750, Santa Cruz Biotechnology, Santa Cruz, CA), mouse anti-ERK1/2 (1:4000, R&D Systems, Wiesbaden-Nordenstadt, Germany), rabbit anti-phospho-Akt^Thr308^ (1:2000, Cell Signaling, Boston, MA) and rabbit anti-phospho-ERK1/2^(Thr202/Tyr204)^ (1:2000, Cell Signaling). After washing (four times, 4 min each) with 1 × TBST, blots were incubated for 1 h at room temperature with the corresponding peroxidase-conjugated secondary antibodies: rabbit anti-goat (1:10000, Calbiochem, Darmstadt, Germany), goat anti-mouse (1:30000, Calbiochem), and goat anti-rabbit (1:20000, Calbiochem). Following subsequent washing steps, the blots were developed using the SuperSignal West Pico Chemiluminescent Substrate (Thermo Scientific, Pierce Technology) according to the manufacturer’s instructions. Pictures were captured, with different exposure times, using a cooled charge-coupled device (CCD) camera (Raytest, Isotopenmessgeraete GmbH, Germany) and analyzed with AIDA 2.11 software (Raytest, Isotopenmessgeraete GmbH).

### Detection of apoptosis with terminal deoxynucleotidyl transferase dUTP nick end labeling staining

Cell death was determined with terminal deoxynucleotidyl transferase dUTP nick end labeling (TUNEL) staining in retinal sections of saline, insulin (12.5 µl 0.3 µM), U0126 (12.5 µl 100 µM), and Ly294002 (12.5 µl 100 µM) injected chicks (as described in Table 1, for groups 1–3). The posterior part of the eyes, without the vitreous body, was fixed for 20 min in 4% paraformaldehyde, and 12-µm-thick vertical sections were taken. Apoptotic cells containing fragmented DNA were identified with the TUNEL method using the In Situ Cell Death Detection Kit, Fluorescein (Roche Applied Science, Mannheim, Germany) according to the manufacturer’s instructions. DN*ase* I-treated retinas (10 min) served as positive control.

### Statistics and data analysis

All data represent mean values from six animals and standard errors of the mean. For refractive states and ocular dimensions, data represent the difference between the end of the experiment and the baseline levels. For western blot analyses, the relative band intensities were normalized to a control sample. This control sample contained a mixture of two untreated retinas, and one lane per gel was always left for this control sample to control for any variation in signal intensities between different blots and to control for contralateral effects. The ratio of the phosphorylated-form/total-form was calculated with the normalized values and converted into percentages. All data were controlled for normal distribution with a Shapiro–Wilk test and exposed to analyses of variance (one-way ANOVA). Using a one-way ANOVA, the effects of drug injections in the no lens, positive lens–, or negative lens–treated eyes were compared. A significant ANOVA (p<0.05) was followed by a Student *t* test for post hoc analysis. Statistical tests were performed using JMP version 8 software (SAS Institute, Cary, NC).

## Results

### Effects of lens wear and intravitreal insulin, U0126, and Ly294002 on refractive development and axial eye growth

#### Effects of intravitreal injections and lens treatment on refractive development

TUNEL staining was employed to control for potential toxicity. However, no apoptotic cells were detected at the doses used for insulin and its inhibitors (data not shown).

**Figure 1:**
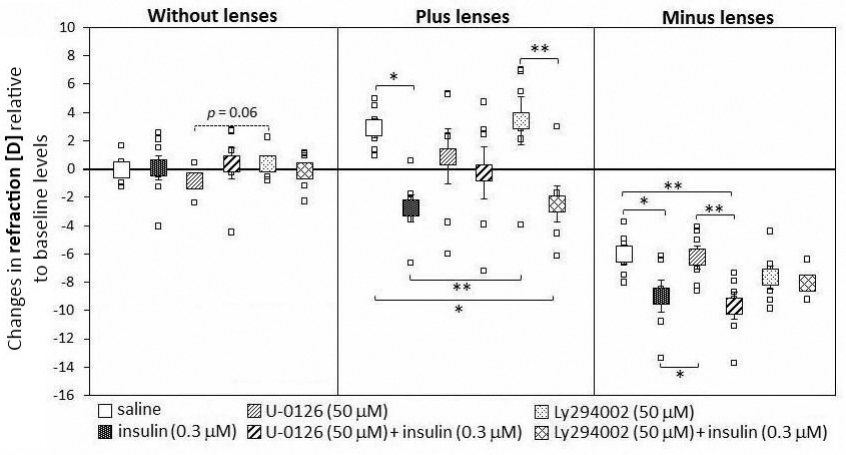
Effects of intravitreal saline, insulin, mitogen-activated protein kinase kinase inhibitor U0126, and phosphatidylinositol 3-kinase inhibitor Ly294002 injections on the development of the refractive state in chicks without any lenses, and after bilateral treatment with +7 **D**: lenses and −7 **D**: lenses. The large squares represent the means of the changes in refractive state±SEM from the beginning of the experiment and four days later. The small squares denote data from individual eyes. Six animals per group were used. Insulin and insulin + PI3K inhibitor prevented hyperopia development in positive lens–wearing animals, whereas the MEK inhibitor blocked the insulin effect. Animals wearing negative lenses and injected with insulin and insulin+ MEK inhibitor overcompensated the imposed defocus. In contrast, with the same lens treatment, the PI3K inhibitor plus insulin–injected eyes had similar refractions as insulin-injected eyes. Statistically significant differences, as determined by one-way ANOVA, are denoted in the graph (* p<0.05, ** p<0.01).

In animals not wearing lenses, none of the injections induced significant changes in the refractive state (Figure 1). There was only a trend (p=0.06) of an opposite effect of both inhibitors: the MEK inhibitor U0126 induced a trend of a myopic shift compared to the PI3K inhibitor Ly294002-injected eyes.

In accordance with previous studies [19,20], insulin-injected animals wearing positive lenses did not become hyperopic but rather more myopic than age-matched controls without injections (Figure 1, Table 2). PI3K inhibitor Ly294002 injections had no effect in animals treated with positive lenses, suggesting that the lenses were compensated as normal. Insulin in combination with this PI3K inhibitor was as effective as insulin alone: it prevented hyperopia (refraction after four days with positive lenses: insulin versus insulin and Ly294002+0.49±0.81 D versus +0.61±1.46 D, Table 2). Interestingly, refractive states in animals injected with the MEK inhibitor U0126 or insulin plus MEK inhibitor were similar after four days of positive lens wear (U0126 versus U-126 plus insulin: +3.88±2.07 D versus +3.00±2.02 D, n.s.), suggesting that this inhibitor blocked the effect of insulin. Standard errors in MEK inhibitor–injected eyes were higher than in all other groups.

**Table 2:** Effects of lens wear and intravitreal insulin, U0126 and Ly294002 injections, after 4 days of treatment, on refractive development and ocular dimensions.

Injection/ Lens treatment	Saline	Insulin (0.3 µM)	U0126 (50 µM)	U0126+insulin (50 µM+0.3 µM)	Ly294002 (50 µM)	Ly294002+insulin (50 µM+0.3 µM)
4 days WITHOUT LENSES (n=6)						
Refraction [D]	+2.79±0.28	+2.91±0.91	+2.28±0.39	+3.50±1.11	+3.90±0.65	+2.98±0.55
ACD [mm]	1.19±0.05	1.17±0.02	1.20±0.02	1.20±0.02	1.17±0.04	1.21±0.03
LT [mm]	2.21±0.04	2.17±0.02	2.10±0.01	2.13±0.03	2.10±0.04	2.17±0.03
VCD [mm]	5.19±0.05	5.06±0.09	5.20±0.03	5.09±0.06	5.20±0.05	5.09±0.08
Axial length [mm]	8.51±0.08	8.40±0.09	8.50±0.03	8.43±0.05	8.47±0.07	8.47±0.08
4 days with PLUS LENSES (n=6)						
Refraction [D]	+6.25±0.79	+0.49±0.81	+3.88±2.07	+3.00±2.02	+6.40±1.64	+0.61±1.46
ACD [mm]	1.02±0.05	1.18±0.05	1.06±0.05	1.21±0.07	1.15±0.06	1.24±0.09
LT [mm]	2.05±0.06	2.20±0.03	2.12±0.05	2.22±0.02	2.11±0.02	2.21±0.03
VCD [mm]	4.94±0.07	5.01±0.07	5.08±0.08	5.01±0.07	5.02±0.07	5.08±0.10
Axial length [mm]	8.01±0.09	8.39±0.10	8.26±0.11	8.44±0.14	8.28±0.10	8.53±0.17
4 days with MINUS LENSES (n=6)						
Refraction [D]	−3.32±0.90	−6.09±1.11	−3.79±1.05	−6.83±0.88	−4.81±0.89	−6.75±1.59
ACD [mm]	1.20±0.06	1.34±0.04	1.23±0.08	1.36±0.04	1.22±0.07	1.35±0.06
LT [mm]	2.14±0.04	2.25±0.05	2.15±0.04	2.30±0.06	2.18±0.08	2.11±0.04
VCD [mm]	5.48±0.10	5.45±0.07	5.51±0.09	5.50±0.12	5.48±0.13	5.55±0.10
Axial length [mm]	8.82±0.12	9.03±0.07	8.88±0.16	9.15±0.15	8.88±0.13	9.03±0.13

Refractive state (in diopters, D) and ocular dimensions (in mm) on day 4 after the lens and injections treatment. Results are represented as mean±SEM. For all groups n=6. ACD represents anterior chamber depth; LT represents lens thickness; VCD represents vitreous chamber depth; AL represents axial length.

In agreement with previous studies [19], animals wearing −7 D lenses and injected with insulin not only became myopic but also overcompensated the negative lenses. The mean change in refraction was −6.01±0.68 D in the saline-injected group and −9.05±1.15 D in the insulin-injected animals, with a difference of 3.04±1.65 D (95% confidence interval [CI], 0.54 D; 5.55 D, p=0.019). This effect was similar when only the MEK inhibitor was injected or the MEK inhibitor in combination with insulin (difference 3.53 D, 95% CI, 1.03 D; 6.04 D, p=0.007). In contrast, insulin plus PI3K inhibitor–injected eyes did not become more myopic than eyes injected with only PI3K inhibitor. In those cases, the eyes compensated for the power of the negative lenses as normal.

### Effects of intravitreal injections on ocular dimensions

**Figure 2:**
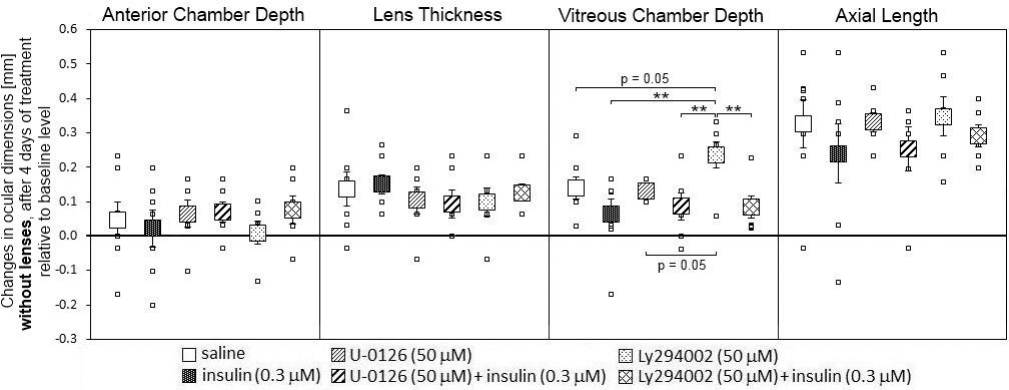
Effects of intravitreal saline, insulin, mitogen-activated protein kinase kinase inhibitor U0126, and phosphatidylinositol 3-kinase inhibitor Ly294002 injections on the ocular compartments in chicks without any lenses. The large squares represent the means of the changes±SEM from the beginning of the experiment and four days later. The small squares denote data from individual eyes. Six animals per group were used. Animals injected with PI3K inhibitor had deeper vitreous chambers compared with all the other types of injections. No significant changes were observed in anterior chamber depth, lens thickness, and axial length. Statistically significant differences, as determined with one-way ANOVA, are denoted in the graph (* p<0.05, ** p<0.01).

In animals not wearing any lenses, the injections had no significant effect on the growth of the anterior chamber, the crystalline lens, and axial length (Figure 2). Vitreous chamber depth (VCD) was significantly longer in Ly294002-injected eyes than in most other injection groups. Nevertheless, this difference in vitreous chamber depth was small (VCD changes: saline: 0.14±0.03 mm; Ly294002: 0.24±0.04 mm) and was not correlated with a change in refraction (see Figure 1).

**Figure 3:**
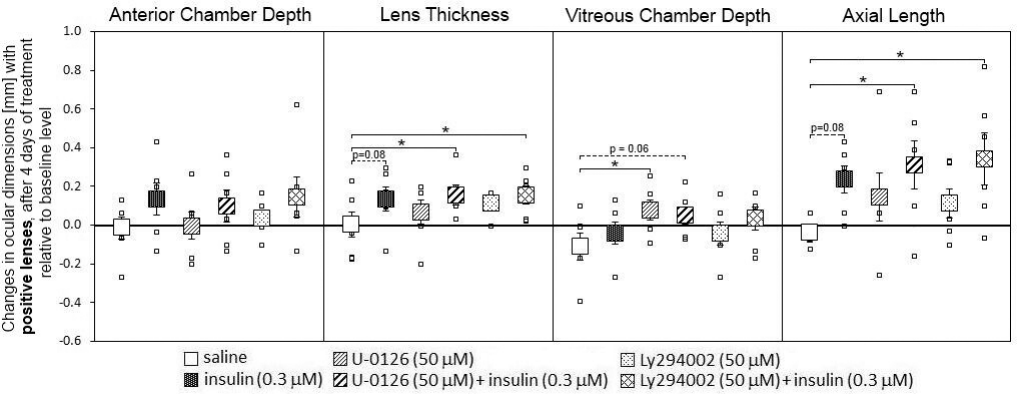
Effects of intravitreal saline, insulin, mitogen-activated protein kinase kinase inhibitor U0126, and phosphatidylinositol 3-kinase inhibitor Ly294002 injections on the ocular compartments in chicks treated with positive lenses during four days. The large squares represent the means of the changes±SEM from the beginning of the experiment and four days later. The small squares denote data from individual eyes. Six animals per group were used. Statistically significant differences, as determined with one-way ANOVA, are denoted in the graph (* p<0.05, ** p<0.01).

No significant changes were detected in the anterior chamber depth (ACD) in animals treated with positive lenses and injected with different drugs (Figure 3). Insulin-injected eyes showed a trend of developing thicker lenses, compared with saline-injected eyes, and this effect was even larger and reached significance in eyes that were injected with insulin in combination with U0126 or Ly294002. The same changes were observed for axial length where the effect of insulin alone was not significant but where insulin in combination with both inhibitors induced a significant increase in axial length compared to saline-injected eyes. Similar to previous studies, positive lens treatment inhibited vitreous chamber growth in saline-injected eyes [19]. When injected with insulin, VCD did not significantly differ from the saline-injected group in this experiment (Figure 3). A significant suppression of the effects of positive lenses on VCD was seen only in animals injected with U0126. Those eyes had significantly longer VCDs than saline-injected eyes (Table 2, VCD after four days of positive lens treatment; saline versus U0126: 4.94±0.07 mm versus 5.08±0.08 mm, p<0.05). The longer axial length in eyes injected with insulin plus Ly294002 compared to saline-injected eyes correlated with a more myopic refraction. However, although the eyes injected with insulin plus MEK inhibitor U-0128 were significantly longer than the saline-injected eyes, their refractions, as shown in Figure 2, were not significantly different from the saline group.

**Figure 4:**
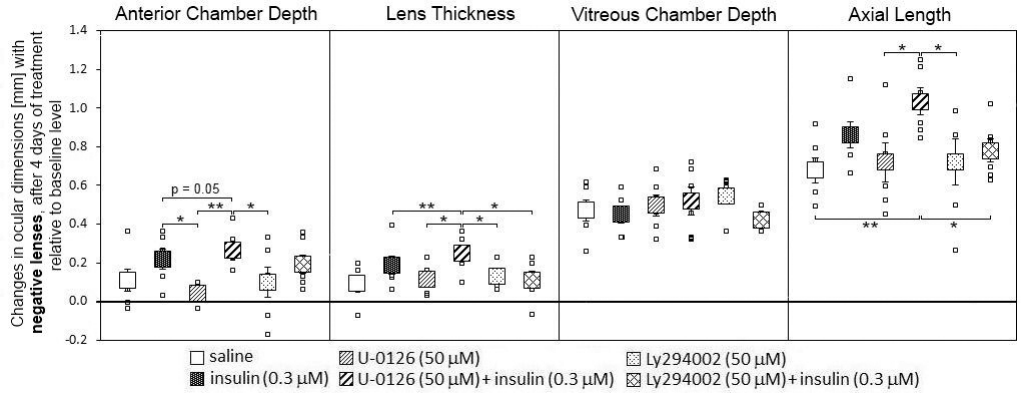
Effects of intravitreal saline, insulin, mitogen-activated protein kinase kinase inhibitor U0126, and phosphatidylinositol 3-kinase inhibitor Ly294002 injections on the ocular compartments in chicks treated with negative lenses during four days. The large squares represent the means of the changes±SEM from the beginning of the experiment and four days later. The small squares denote data from individual eyes. Six animals per group were used. Statistically significant differences, as determined with one-way ANOVA, are denoted in the graph (* p<0.05, ** p<0.01).

Animals treated with negative lenses and injected with a combination of insulin and the MEK inhibitor U0126 had significantly deeper ACDs, thicker crystalline lenses, and longer axial lengths, compared with animals injected with U0126 or Ly294002 alone (Figure 4). In addition, they had a trend of a longer ACD compared with insulin-injected animals (p=0.05), a thicker lens compared with the eyes injected with insulin and insulin plus Ly294002, and longer axial length than the eyes injected with saline and Ly294002 plus insulin. The highest axial length in the eyes injected with insulin plus U0126 correlated with the highest amount of myopia measured in this group (see Figure 1). However, the injections had no significant effect on the growth of the VCD in minus lens–treated animals.

### Effects of short- and long-term lens wear, combined with intravitreal injections of insulin, U0126, and Ly294002 on extracellular regulated kinase 1/2 and protein kinase B phosphorylation levels

**Figure 5:**
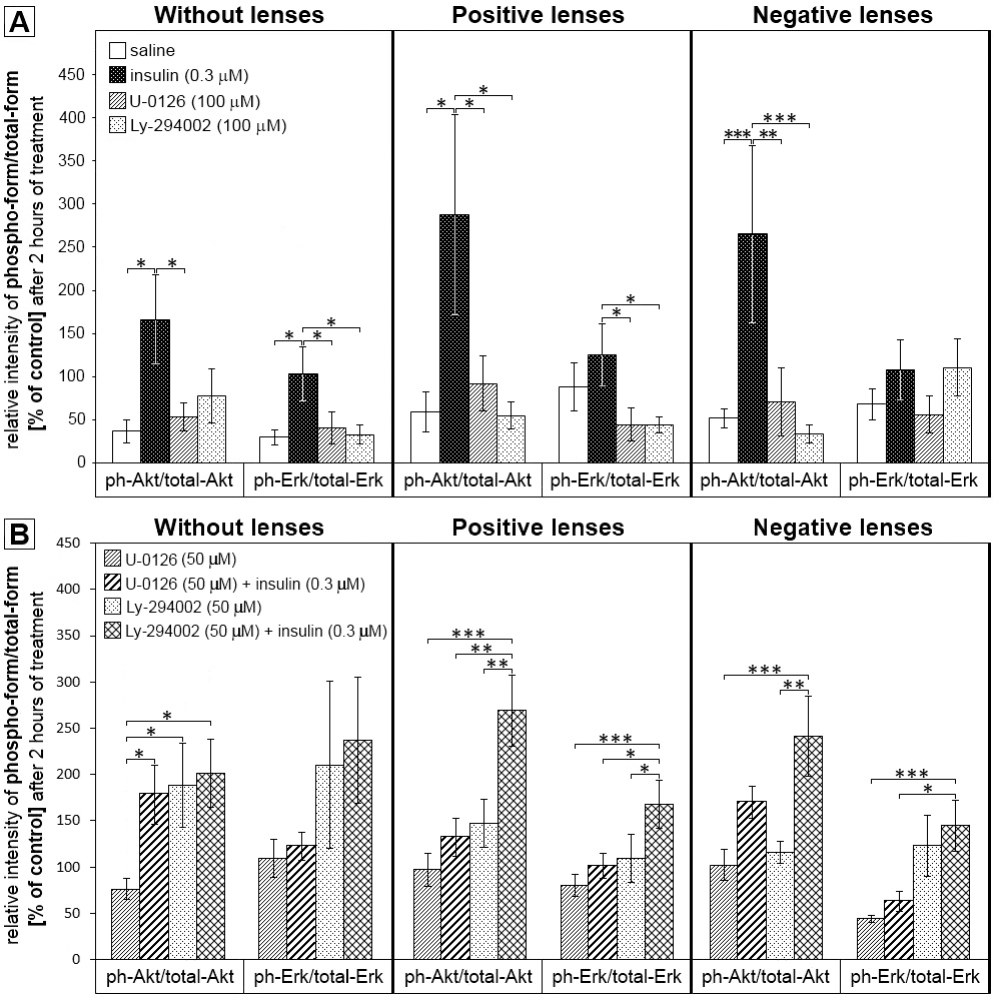
Short time effects (2 h) of intravitreal saline, insulin, mitogen-activated protein kinase kinase inhibitor U0126, U0126 plus insulin, phosphatidylinositol 3-kinase inhibitor Ly294002, or Ly294002 plus insulin injections on phospho-Akt/total-Akt and phospho-Erk/total-Erk levels in chicks not wearing any lenses, and after bilateral treatment with +7 **D**: lenses and −7 **D**: lenses. The same independent control sample was analyzed on each blot and used for normalization of the data. Band intensities were converted to percentages of the control sample value. The ratio of phospho-form/total-form was then calculated using the normalized values. The bars represent the mean ratio of relative intensity (mean±SEM) between the phospho-forms and non-phosphorylated proteins in percent. Each group included six animals. In control, positive and negative lens–wearing animals (Figure 5A) insulin injections highly increased the retinal ratio of phospho-Akt/total-Akt. In control and animals wearing positive lenses, insulin injections led to an increase in phospho-Erk/total-Erk levels, compared to either the MEK inhibitor or the PI3K inhibitor (Figure 5A). The PI3K inhibitor blocked the stimulatory effect of insulin in control animals without lenses but not in positive and negative lens–treated animals (Figure 5B). The MEK inhibitor significantly blocked the effect of insulin on ERK phosphorylation (Figure 5B). Significant differences were determined with one-way ANOVA and are denoted in the graph (* p<0.05, ** p<0.01, and *** p<0.001).

Western blots were used to find out whether insulin activates the MEK/ERK and/or PI3K/Akt pathway in the retina. A second question was whether lens treatment per se can activate one of those pathways. The MEK inhibitor U0126 and the PI3K inhibitor Ly294002 were used alone (Figure 5A) or in combination with insulin (Figure 5B).

As shown in Figure 5A, intravitreal insulin injections stimulated both pathways. The retinal level of phospho-Akt/total-Akt was significantly elevated, compared to the saline-injected and MEK inhibitor–injected eyes in animals without lens treatment (Figure 5A). The insulin-induced activation of Akt was even more pronounced in animals wearing positive lenses and negative lenses. Here, insulin caused a more than threefold increase in phospho-Akt/total-Akt levels, compared to all other injection groups. Insulin activated not only the PI3K/Akt pathway but also, to a lesser extent, the MEK/ERK pathway. In animals without lenses, significant changes in phospho-ERK/total-ERK levels were induced by insulin compared with all other injection groups. Insulin injections also led to a significant increase in phospho-ERK/total-ERK levels in animals wearing positive lenses, compared to U0126- and Ly294002-injected eyes (Figure 5A). In contrast, no insulin-induced activation of this pathway was detected in negative lens–treated eyes (Figure 5A). The inhibitors U0126 and Ly294002 alone did not significantly influence the retinal level of phospho-Akt and phospho-ERK compared to saline-injected eyes in animals without lenses or in chicks treated with positive and negative spherical lenses (Figure 5A). In addition, positive and negative lens treatment per se did not significantly alter the amount of phospho-Akt/total Akt or of phospho-ERK/total ERK (one-way ANOVA, saline injections: no lens wear versus plus lens wear versus minus lens wear, n.s.).

Furthermore, insulin was injected in combination with either U0126 or Ly294002 to test the efficiency and specificity of the inhibitors in vivo (Figure 5B). We already knew from our studies (Figure 5A) that insulin stimulated the PI3K/Akt pathway in chicks without lenses, as well as in chicks with positive lenses, and with negative lenses. We therefore expected that the same amount of phospho-Akt/Akt would be found in eyes injected with PI3K inhibitor plus insulin, compared to the inhibitor-injected fellow eyes, as long as the inhibitor worked. Inhibitor concentrations were chosen based on a study by Chavarria et al. in organotypic chicken retinal cell cultures [50]. With no lens treatment, the amount of phospho-Akt/Akt in the Ly294002-injected eyes versus the insulin plus Ly294002-injected eyes was nearly identical, showing that the PI3K inhibitor blocked the stimulatory effect of insulin (Figure 5B, third and fourth bars). In contrast, Ly294002 did not block the effects of insulin in positive and negative lens–treated animals.

Based on our results (Figure 5A), we also expected to see the same amount of phospho-EKR/ERK in the groups injected with MEK inhibitor plus insulin compared to their contralateral insulin-injected eyes in animals with no lenses, or with positive lenses, if the inhibitor worked. Indeed, the MEK inhibitor U0126 blocked the effect of insulin on ERK phosphorylation (Figure 5B, fifth versus sixth bar, 13^th^ versus 14^th^ bar). Since insulin did not stimulate phosphorylation of ERK in the negative lens–treated group (Figure 5A), we could not study the potency of the MEK inhibitor in those retinas.

An effect of the PI3K inhibitor on insulin-induced ERK phosphorylation and of the MEK inhibitor on insulin-induced Akt phosphorylation was not expected, unless both pathways interacted and controlled the phosphorylation level of each other’s components. This was indeed found in some groups. The MEK inhibitor U0126 did not block the insulin-induced increase in Akt phosphorylation in eyes without lenses (Figure 5B, first and second bars). This result therefore does not support a cross-talk between the pathways. In contrast, the MEK inhibitor blocked the insulin effect on Akt phosphorylation in plus and minus lens–treated animals (Figure 5B: phosphoAKt/Akt, plus lens, U0126 versus insulin plus U0126: n.s., U0126 versus insulin plus Ly294002: p<0.001). The MEK inhibitor was even more potent than the PI3K inhibitor itself (Figure 5B: phosphoAkt/Akt, plus lens: insulin plus U0126 versus insulin plus Ly294002, p<0.01). The potency of the PI3K inhibitor on ERK phosphorylation depended on the lens treatment. The PI3K inhibitor Ly294002 did not block the insulin-induced increase in ERK phosphorylation in eyes treated with plus lenses but in animals without lenses.

After four days of lens treatment, and injections every other day, the retinal levels of phospho-Akt/total-Akt and phospho-ERK/total-ERK were not significantly different and seemed to have a ceiling (data not shown).

## Discussion

After ligand binding, the insulin receptor can recruit and phosphorylate IRS proteins. Depending on the stimuli and the phosphorylated IRS, two different pathways are activated: the PI3K/Akt and MEK/ERK pathways. Although intravitreal insulin [19,20] and insulin-like growth factor-1 (IGF-1) [20] can induce myopia in chicks, it is not known if both or only one of the two signaling pathways is responsible for this effect. Therefore, in the present study, we attempted to find out how inhibiting either pathway affects eye growth. The effects of insulin and two specific pathway inhibitors were investigated under different visual conditions (normal vision or treatment with positive or negative lenses).

The current study showed that insulin and PI3K and MEK inhibitors induced only small changes in refractive states in eyes with normal visual exposure. Both pathways were activated because the levels of phospho-Akt/Akt and phospho-ERK/ERK were significantly increased in insulin-injected eyes, and the specific inhibitors blocked these stimulatory effects of insulin.

As soon as lenses were used to defocus the retinal image, much larger effects of insulin on refractive development and on the biometry of the eye were seen. Insulin prevented the compensation of positive lenses, in line with previous findings [19]. Although the PI3K inhibitor did not block this effect of insulin, the MEK inhibitor did. This suggests that the effect of insulin on positive lens compensation is likely to be mediated by activation of the MEK/ERK pathway. The effects of the MEK inhibitor on the development of the refractive state were correlated with high amounts of phospho-ERK/total-ERK in the insulin-injected, positive lens–treated eyes compared to the PI3K or MEK inhibitor–injected eyes and with an effective block of ERK activation in eyes in which insulin was injected together with the MEK inhibitor. The changes were seen in western blots after 2 h, emphasizing the importance of this pathway when positive lenses are compensated. In addition, the PI3K/Akt pathway was activated in plus lens–treated animals, but the PI3K inhibitor did not completely block this activation.

In agreement with previous studies [19], insulin induced not only myopia but also overcompensation of the power of the lenses in animals wearing negative lenses. Only the PI3K inhibitor reduced the insulin-induced overcompensation. Western blots revealed that insulin did not significantly influence the MEK/ERK pathway but highly increased the amount of retinal phospho-Akt/total-Akt in animals treated with negative lenses for 2 h. These results emphasize the importance of the PI3K/Akt pathway when positive defocus is imposed and insulin concentrations are high. Although the PI3K inhibitor reduced the insulin effect in minus lens–treated chicks, the inhibitor did not completely block the activation/phosphorylation of Akt. Therefore, the doses used in our study may not have been high enough to completely block the pathway. There may also be a direct interaction between both pathways because the MEK inhibitor was more potent in decreasing the phosphorylation of Akt in plus and minus lens–treated animals than the PI3K inhibitor itself.

Changes in refractive errors did not always correlate with changes in biometry. In particular, insulin injections caused a similar increase in axial length and lens thickness in combination with both inhibitors in plus lens–treated animals, but the refractions were different. High variability in the ocular components might have combined to different refractive outcomes, in particular in the data of the MEK inhibitor–injected eyes. In addition, changes in corneal curvature were not considered. In animals treated with negative lenses, the refractive changes were in general in agreement with the changes observed in the biometry of the eyes. The group that developed the highest amount of myopia (insulin plus MEK inhibitor injection) also had the longest axial length, the deepest anterior chamber, and the thickest lens.

In summary, the current study may show that insulin activates the PI3K/Akt pathway in eyes with normal vision and eyes treated with positive and negative lenses but stimulates eye growth through the MEK/ERK pathway in eyes with normal vision and eyes treated with positive lenses. As has been concluded from studies of other signaling pathways, the current study adds support to the idea that positive and negative lenses activate different biochemical pathways.

Based on our findings, we propose the following hypothesis. Since Egr-1 is an immediate early gene that responds to defocus of different sign in opposite directions and has an important role in emmetropization [19,24,26,27], we assume that the changes represent the initial event when changes in eye growth occur. We propose that Egr-1 controls the balance between the PI3K/Akt and MEK/ERK pathways. There is already evidence from other systems that the preferential activation of the PI3K/Akt and MEK/ERK pathways is mediated by Egr-1. In these studies, Egr-1 was hypothesized to be an important regulator of insulin sensitivity by controlling the balance between these pathways in mice [56,57]. Intravitreal injections of insulin had powerful stimulatory effects on Egr-1 mRNA levels in eyes with normal vision and eyes treated with positive lenses. There was increased Egr-1 protein expression in Müller cells and bipolar cells [19]. Furthermore, insulin reduced the number of ZENK-positive glucagon amacrine cells in positive lens–treated eyes. This is opposite of what is normally observed in eyes with positive lenses but seen in eyes that develop myopia.

Recent studies showed that under hyperinsulinism stress, Egr-1 is able to control the balance between the Akt and MAPK pathways in fat tissue by inducing the expression of downstream genes involved in the pathways. Sustained expression of Egr-1 can stimulate phosphatase and tensin homolog deleted on chromosome 10 (*PTEN*) and geranylgeranyl diphosphate synthase (*GGPPS*) transcription [56]. Increased *PTEN* blocks PI3K/Akt signaling and thus impairs insulin-induced glucose metabolism. Meanwhile, increased *GGPPS* causes sustained ERK1/2 activation. If the same mechanism operated in the eye, this would support our hypothesis that eye growth induced by insulin injections follows a change in the Egr-1-mediated balance between the PI3K/Akt and MEK/ERK pathways. Since we know that positive lens treatment also leads to an increase in Egr-1, this could cause sustained ERK activation.

In addition, other downstream molecules from both pathways need to be studied under different visual conditions to confirm our hypothesis. A microarray study [58] showed that 24 h of positive lens treatment induced upregulation of growth factor receptor-bound protein 2 (*Grb2*), which is implicated in the insulin pathway, by activating the MAPK pathway through the interaction with Ras and the son of sevenless [59]. In that study, the authors found upregulation of *Grb2* in negative and positive lenses–treated animals although the upregulation was stronger with negative lenses.

Cordain et al. [60,61] proposed that a change in nutrition toward a higher intake of refined carbohydrate and sugar might be responsible for the dramatic increase in myopia: diets high in refined carbohydrates are increasing glucose levels (hyperglycemia) and consequently insulin levels (hyperinsulinemia), and insulin resistance, which, in turn, induces growth of the eyeball. One could speculate that a sustained hyperinsulinism, stimulated by insulin and enhanced by insulin resistance [56], could impair the balance between PI3K/Akt and MEK/ERK signaling.

### CONCLUSION

Our study suggests that insulin stimulates the PI3K/Akt pathway under all visual conditions. In contrast, the MEK/Erk pathway was preferentially stimulated only in chicks with normal visual exposure and in chicks treated with positive spectacle lenses. The MEK/Erk pathway was not stimulated in chickens treated with negative spectacle lenses. Identifying key regulators that control the balance between the two signaling pathways may provide a new strategy for pharmacological intervention of myopia.
